# CMR-derived left atrial reservoir strain and the LAVI/LVEF ratio are associated with 30-day readmission in atrial fibrillation: the CMRRADAR-AF retrospective cohort study

**DOI:** 10.3389/fcvm.2026.1845659

**Published:** 2026-10-20

**Authors:** Maethas Panyawongkhanti, Anuchai Fueangtalop, Nattanon Satitthammaphon, Chokanan Thaitirarot, Viroj Mueangsilpsat, Chanwit Wuttichaipradit, Anusith Tunhasiriwer, Chonthicha Tanking

**Affiliations:** 1 Princess Srisavangavadhana Faculty of Medicine, Chulabhorn Royal Academy, Bangkok, Thailand; 2 Department of Cardiovascular Medicine, Chulabhorn Hospital, Bangkok, Thailand; 3 Department of Radiology, Chulabhorn Hospital, Bangkok, Thailand; 4 Department of Cardiology, Chulabhorn Hospital, Bangkok, Thailand

**Keywords:** atrial fibrillation, atrial hyperenhancement, cardiovascular magnetic resonance, CVI42, LA reservoir strain, LACE score, LAVI/LVEF ratio, readmission

## Abstract

**Background:**

Cardiovascular magnetic resonance (CMR) imaging allows simultaneous evaluation of cardiac structure, function, and tissue composition. Whether CMR-derived left atrial (LA) reservoir strain, the left atrial volume index–left ventricular ejection fraction (LAVI/LVEF) ratio, and atrial late gadolinium enhancement (LGE) can identify patients at risk for 30-day readmission after atrial fibrillation (AF)–related hospitalisation has not been investigated.

**Objectives:**

This study aims to evaluate the association between CMR parameters and 30-day unplanned readmission following an AF-related index admission and to describe CMR differences across three clinical groups using a three-group cohort design.

**Methods:**

This retrospective cohort study at Chulabhorn Hospital, Bangkok (IRB005/2569), enrolled 43 patients with AF who underwent CMR on 1.5 T or 3 T Philips scanners: 25 outpatients (OPD), 10 index-admitted patients without readmission (Adm-NoReadmit), and 8 index-admitted patients with 30-day unplanned readmission (Readmit). A CMR analysis was performed using CVI42 version 6.4. LA reservoir strain was derived by feature tracking from standard cine images.

**Results:**

Among the admitted patients (*n* = 18), LA reservoir strain showed the highest apparent discrimination for readmission [area under the curve (AUC), 0.963; *p* < 0.001; exploratory internally derived cutpoint <11.1%: sensitivity, 88%; 95% confidence interval (CI), 47%–100%; specificity, 100%; 95% CI, 69%–100%]. Correcting for predictor selection, the optimism-corrected AUC was 0.919. The LAVI/LVEF ratio was also associated with readmission (AUC, 0.913; *p* = 0.002). Qualitative atrial hyperenhancement on non-dedicated LGE imaging completely separated readmitted from non-readmitted admitted patients (8/8 vs. 0/10); however, because it was assessed on an LV-optimised protocol not validated for atrial tissue, we report it only as an exploratory qualitative finding and do not interpret it as evidence of a fibrosis mechanism. The LACE score (length of stay, acuity of admission, Charlson comorbidity index, and emergency department visits) achieved an AUC of 0.719 (95% CI, 0.44–0.95; *p* = 0.125); an exploratory combined LACE+ LA reservoir strain model produced a numerically higher apparent AUC, but with only eight events, this estimate is unstable and likely overfitted and is not interpreted as evidence of incremental value. A three-group analysis revealed a non-monotonic pattern: The Adm-NoReadmit patient group had the highest LA reservoir strain (15.4%), exceeding the OPD group (8.5%), whereas the Readmit group had the lowest (6.6%).

**Conclusion:**

In this exploratory feasibility analysis, CMR-derived LA reservoir strain and the LAVI/LVEF ratio were associated with 30-day unplanned readmission following an AF-related index admission. Because readmitted patients had a substantially higher prevalence of heart-failure history, these associations may reflect a shared structural–functional substrate rather than independent, AF-specific effects. Descriptive comparison across the three clinical groups showed a non-monotonic LA functional pattern, suggesting that CMR may aid risk stratification at discharge. However, prospective validation is needed.

## Graphical Abstract

**Figure:**
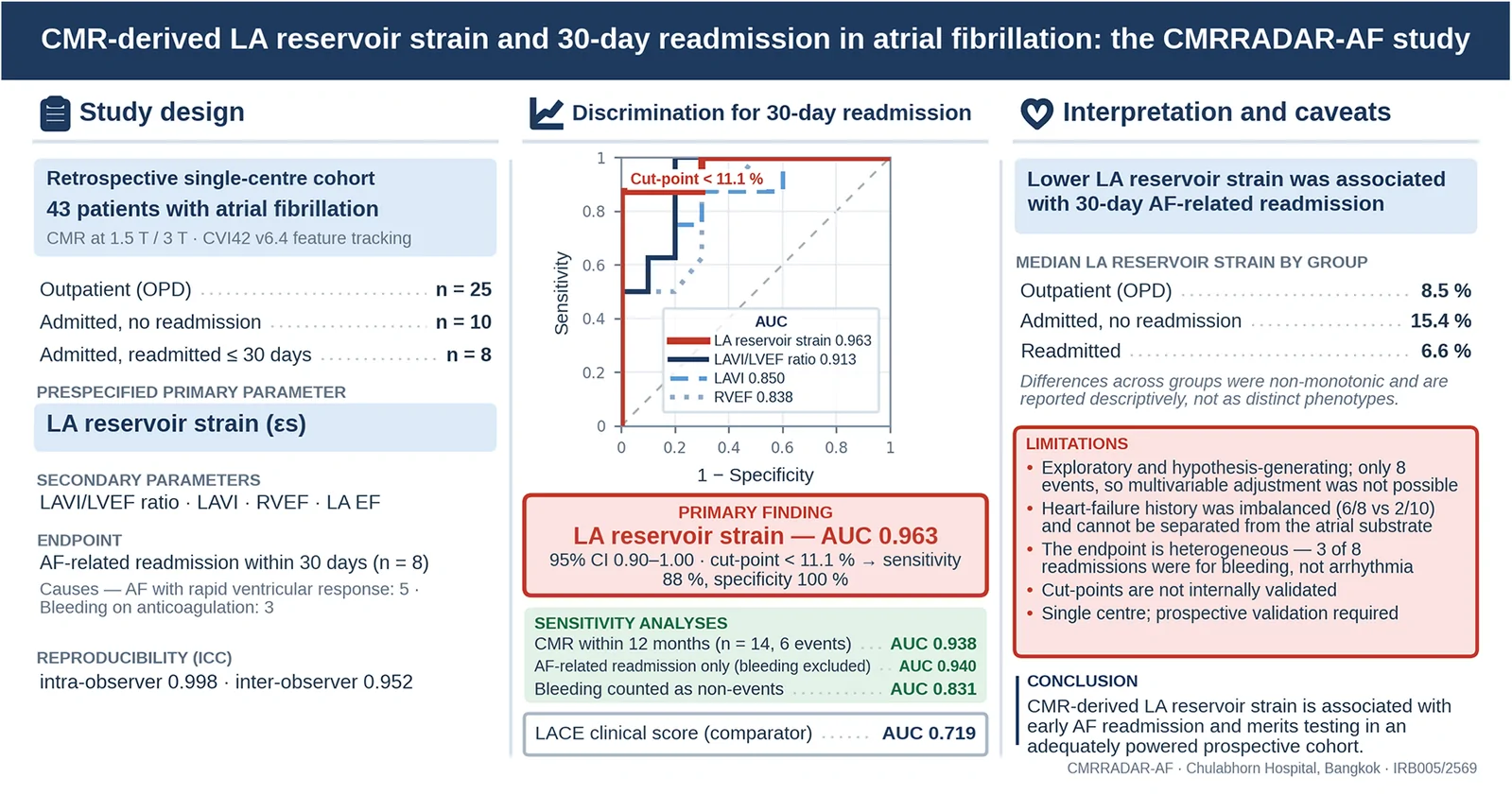
CMR-derived LA reservoir strain and 30-day readmission in atrial fibrillation: the CMRRADAR-AF study.

## 1 Introduction

Atrial fibrillation (AF) is the most common sustained cardiac arrhythmia that affects an estimated 33.5 million people worldwide (2010 estimate), with the risk increasing with age (1). Its clinical impact includes heart failure, thromboembolic stroke, and repeated hospitalisations, all adding to costs and burden (2).

Unplanned 30-day readmission after an AF-related admission is a growing marker of care quality and disease control, indicating unresolved arrhythmic substrate or suboptimal discharge planning (21, 24). Existing risk scores—LACE (length of stay, acuity of admission, comorbidity, and emergency department visits), HOSPITAL, and disease-specific models—only moderately discriminate [area under the curve (AUC), 0.60–0.75] (11, 22, 23) because they rely on clinical and administrative data and miss the structural and functional cardiac substrate driving AF recurrence.

Cardiovascular magnetic resonance (CMR) analyzes cardiac chamber volumes, tissue composition, and myocardial deformation in a single test (3). Left atrial (LA) reservoir strain, obtained by feature tracking (FT), shows how well the left atrial wall stretches during ventricular systole while the mitral valve stays closed (4). Because it depends on ventricular systole and not atrial electrical activity, LA reservoir strain is the most suitable LA strain component in patients with AF who have irregular or no atrial contraction (5).

Atrial late gadolinium enhancement (LGE) can detect and, with dedicated protocols, quantify atrial fibrosis—an established substrate for AF persistence and recurrence—as shown in the DECAAF trials (6). Another parameter of interest is the left atrial volume index–left ventricular ejection fraction (LAVI/LVEF) ratio, a simple composite volume-to-function measure [distinct from the validated left atrioventricular coupling index, which is defined as LA end-diastolic volume divided by left ventricle (LV) end-diastolic volume] (26). This ratio was initially described by Yilmaz et al. as an independent correlate of major adverse cardiovascular events in patients with ST segment elevation myocardial infarction (STEMI) over an 8-year follow-up (7) and has since been associated with adverse outcomes in transthyretin cardiac amyloidosis (8). Its performance in AF readmission has not been evaluated.

The objectives of this study were to (1) determine whether CMR parameters are associated with 30-day unplanned readmission following an AF-related index admission; (2) describe CMR differences across three clinical groups spanning the spectrum from stable outpatient AF to recurrent hospitalisation; and (3) assess the LAVI/LVEF ratio (a simple composite volume-to-function measure, distinct from the validated left atrioventricular coupling index) in this setting.

## 2 Methods

### 2.1 Study design and population

This was a single-centre, retrospective, observational cohort study conducted at Chulabhorn Hospital, Bangkok, Thailand. Patients were identified through electronic medical records from January 2018 to December 2024. Inclusion criteria were given as follows: (1) age ≥18 years, (2) documented AF (International Classification of Diseases, 10th Revision, ICD-10, codes I48.0–I48.9), and (3) available CMR data acquired within 5 years of clinical evaluation. Patients were excluded if they had no AF-related clinical episode during the study period, lacked key demographic data, or had undergone CMR more than 5 years before the index clinical event. This study was approved by the Institutional Review Board of Chulabhorn Royal Academy (IRB005/2569) and conducted in accordance with the Declaration of Helsinki. Of 1,298 patients identified with AF by ICD-10 codes, 158 had an inpatient AF-related admission; among them, 21 had undergone CMR, 2 were excluded because they had undergone CMR more than 5 years before admission, and 1 was excluded because of an incomplete image dataset, leaving 18 admitted patients. From the outpatient AF population, 28 had undergone CMR, of whom 3 were excluded for incomplete datasets, leaving 25 outpatients (OPD). Enrolment was consecutive (Figure 1).

**Figure 1:**
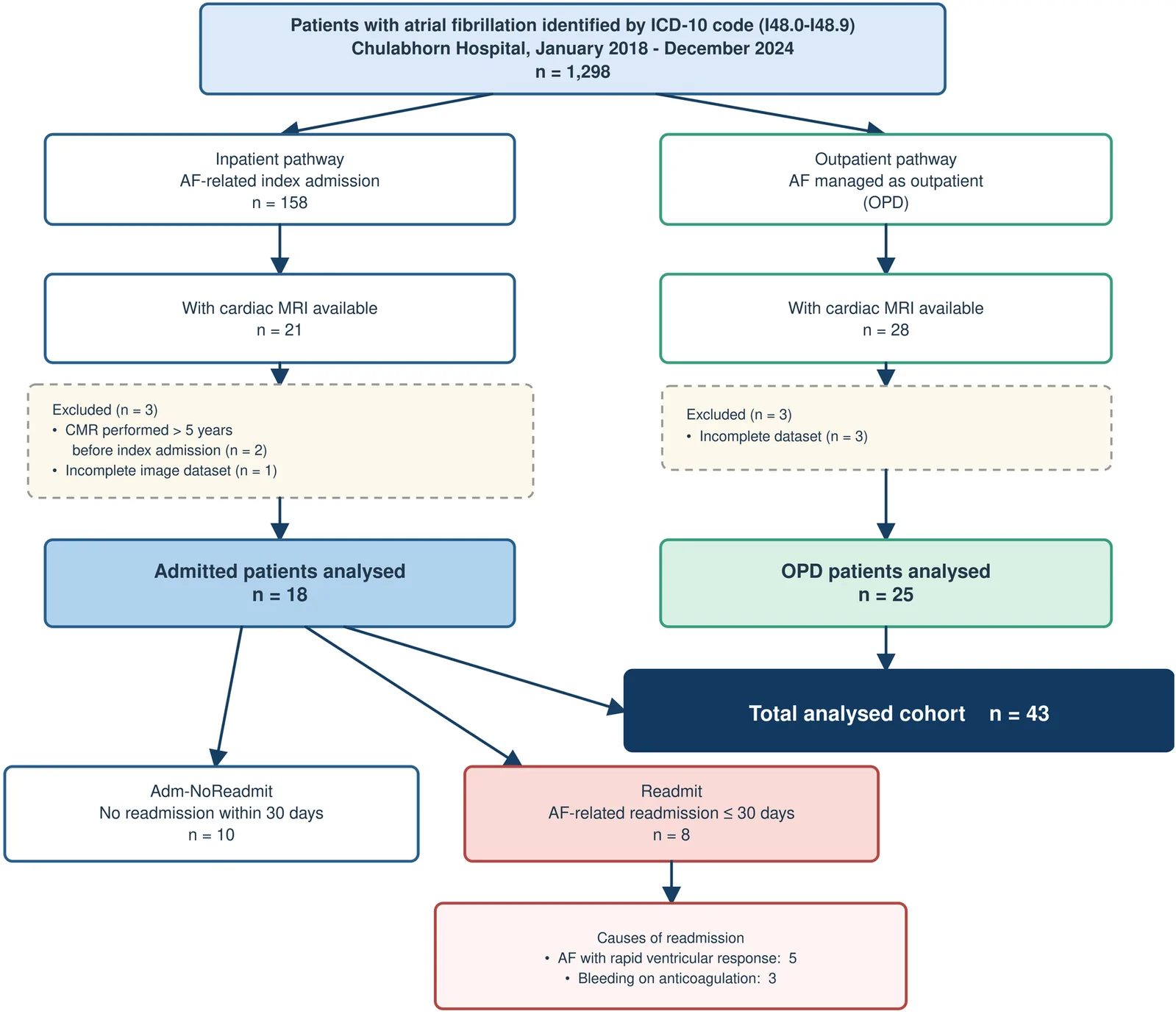
A study flow diagram.

Three clinical groups were defined *a priori*: (1) OPD (patients with AF managed as outpatients without hospitalisation), (2) Adm-NoReadmit (patients admitted for AF-related causes who were not readmitted within 30 days of discharge), and (3) Readmit (patients admitted for AF-related causes who were readmitted within 30 days).

### 2.2 CMR acquisition

CMR was performed on 1.5 T or 3 T Ingenia Philips scanners (Philips Healthcare, Best, the Netherlands) using a dedicated cardiac surface coil with ECG gating. Balanced steady-state free precession (bSSFP) cine imaging was acquired in standard long-axis orientations (two-, three-, and four-chamber views) and a short-axis stack covering both ventricles. Typical acquisition parameters were given as follows: flip angle = 45°–60°, TE = 1.2–2.0 ms, TR = 3.0–4.0 ms, slice thickness = 8 mm, and temporal resolution = 25–30 phases per cardiac cycle.

Post-processing was performed using CVI42 software version 6.4 (Circle Cardiovascular Imaging Inc., Calgary, Canada). LV and right ventricle (RV) volumes, ejection fractions, stroke volumes, cardiac output, and mass were derived from semi-automated contouring of the short-axis stack. LA volume was measured using the biplane area–length method from two- and four-chamber cine images at the end systole and indexed to the body surface area (LAVI, mL/m^2^).

### 2.3 LA reservoir strain measurement

LA reservoir strain was measured by CMR-FT within CVI42 version 6.4, following the methodology described by Hopman et al. and Akkuzu et al. (5, 9). Endocardial and epicardial LA borders were manually traced in both two- and four-chamber long-axis cine views. The LV end diastole was used as the reference frame (zero-strain point), in accordance with the *P* wave–independent convention appropriate for patients with AF. Feature tracking was then applied automatically by the software across all cardiac phases.

LA reservoir strain (εs) represents the peak positive longitudinal strain measured from the LV end diastole to the point of maximum LA volume during ventricular systole. Because this measurement is defined entirely within ventricular systole, it does not require organised atrial electrical activity and is therefore the only LA strain component that is reliably measurable during AF (5). LA booster pump strain (*ε*a) and conduit strain (*ε*e) were not reported, as their derivation requires identification of the atrial contraction phase or the mitral valve opening point, neither of which can be reliably determined during AF.

### 2.4 Late gadolinium enhancement

LGE imaging was performed 10–15 minutes after intravenous gadolinium administration (dose, 0.15–0.175 mmol/kg of body weight) using a phase-sensitive inversion recovery (PSIR) gradient-echo sequence. The inversion time (TI) was individually determined by a preceding Look–Locker scout sequence and set to neutralise the signal of the LV myocardium, consistent with standard clinical practice for ventricular scar assessment. It should be noted that this LV-optimised TI is not specifically tailored to the atrial wall; dedicated atrial LGE protocols (which typically employ shorter TIs targeting atrial myocardium nulling, higher spatial resolution, and respiratory-gated 3D acquisitions) were not used in this study, which represents a methodological limitation for atrial tissue assessment. LV LGE patterns were classified as ischaemic (subendocardial or transmural) or non-ischaemic (midwall, subepicardial, or patchy) using the American Heart Association (AHA) 17-segment model.

Qualitative atrial hyperenhancement was defined as any discrete area of focal hyperenhancement within the LA wall, with signal intensity visually exceeding the adjacent nulled LV myocardium—identifiable on at least one standard LGE plane (two-chamber, four-chamber, or short-axis)—and not attributed to cardiac motion artefact, pulmonary vein inflow, or fat signal. This binary classification (present vs. absent) was adopted because the LV-optimised LGE protocol used in this study does not support reliable quantitative Utah staging of atrial fibrosis burden. All LGE assessments were performed independently by two CMR-trained cardiologists (blinded to clinical group assignment and outcome), and any discordance was resolved by a consensus discussion.

### 2.5 Outcome definition

The primary outcome was 30-day unplanned readmission following an AF-related index hospitalisation, defined as any unplanned hospitalisation within 30 days of index discharge. Anticoagulation-related bleeding was pre-specified as an AF management–related readmission because anticoagulation is a direct consequence of guideline-mandated AF stroke prevention, although it has no atrial substrate mechanism; a pre-specified AF-specific sensitivity analysis excluded these bleeding events. The readmission status was adjudicated by two independent investigators from electronic medical records. Because ascertainment relied on the single-centre electronic medical record, readmissions to other institutions could not be captured, which may under-ascertain events and misclassify the readmission status (see Section 4.6).

### 2.6 CMR-to-index admission interval

For each admitted patient, the interval between the CMR study and the index admission was calculated. All CMR examinations were confirmed to have been performed before the index admission; no post-admission or post-readmission imaging was included. A pre-specified sensitivity analysis was conducted, restricting the cohort to patients whose CMR was performed within 12 months before the index admission.

### 2.7 Reproducibility

Measurement reproducibility was assessed in a subset of 22 CMR examinations. A second CMR-trained reader, blinded to the original values and the clinical group, independently remeasured LA reservoir strain, LAVI, and LVEF and reread the binary atrial hyperenhancement status (inter-observer agreement). The original reader repeated all measurements after an interval of at least 2 weeks while being blinded to the initial values (intra-observer agreement). Agreement for continuous variables was quantified with the intraclass correlation coefficient (ICC; two-way random-effects, absolute agreement, single measure) and for the binary hyperenhancement read with Cohen's *κ*.

### 2.8 Statistical analysis

Analyses were performed using IBM SPSS Statistics version 29. Continuous variables were presented as mean ± SD or as median [interquartile range (IQR))] based on normality testing using the Shapiro–Wilk test. For the primary binary analysis (Readmit vs. Adm-NoReadmit; *n* = 18), continuous variables were compared using an independent-samples t-test or the Mann–Whitney U-test; categorical variables were compared using Fisher's exact test.

For the three-group comparison (*n* = 43), the Kruskal–Wallis test was used for overall group differences, with pairwise Mann–Whitney U *post-hoc* tests and Bonferroni correction. A receiver operating characteristic (ROC) curve analysis was performed for significant continuous CMR parameters, with optimal cutpoints identified by the Youden index. Bootstrap 95% confidence intervals (CIs; 5,000 iterations) were calculated for AUC values. Pairwise AUC comparisons used DeLong's method (10); three pre-specified comparisons were performed: (1) LA reservoir strain vs. LAVI/LVEF ratio, (2) LAVI/LVEF ratio vs. LAVI alone, and (3) LA reservoir strain vs. LAVI alone. For binary predictors, sensitivity and specificity were derived directly from 2 × 2 contingency tables. Qualitative atrial hyperenhancement was assessed on an LV-optimised LGE protocol not validated for atrial tissue, and therefore, it is reported only as an exploratory qualitative finding without a discrimination statistic; similarly, non-ischaemic LV scar is reported descriptively.

To examine intercorrelation among CMR predictors, Spearman’s rank correlation coefficients were calculated for key parameters (LA reservoir strain, LAVI, LAVI/LVEF ratio, left atrial ejection fraction (LAEF), and right ventricular ejection fraction (RVEF)) among the admitted patients. Univariate logistic regression estimated odds ratios per 1-SD increment; Nagelkerke R^2^ was reported as the model fit. To address quasi-complete separation, univariate odds ratios were re-estimated with Firth's penalised-likelihood logistic regression with profile-likelihood confidence intervals. Given the small number of events (*n* = 8), multivariable regression was not performed. The primary analysis was not pre-registered, and no multiplicity adjustment was applied. All ROC-derived cutpoints were reported only as exploratory, internally derived thresholds rather than clinically applicable cutoffs, and sensitivity, specificity, positive predictive value (PPV), and negative predictive value (NPV) were accompanied by exact (Clopper–Pearson) 95% confidence intervals. Because the predictors and cutpoints were selected within the same small sample, an optimism-corrected AUC for the best-performing predictor was estimated by a bootstrap (2,000 resamples) that repeated the entire predictor-selection procedure within each resample. Statistical significance was defined as two-tailed *p* < 0.05.

LACE scores were obtained from the electronic medical records of all 18 admitted patients. The LACE index sums four items: days of hospital stay, whether the admission was emergent (vs. elective), the Charlson Comorbidity Index, and the number of emergency department visits in the preceding 6 months (11). Its predictive performance for 30-day readmission was evaluated by an ROC analysis (bootstrap 95% CI, 5,000 iterations), and the AUC was compared against CMR parameters using a bootstrap-based approach equivalent to DeLong's test. An exploratory additive model combining LACE with LA reservoir strain was examined descriptively; because of the small number of events, no incremental-value metric (such as net reclassification improvement) was relied upon, and this combined analysis is reported only as an exploratory finding.

## 3 Results

### 3.1 Study population and baseline characteristics

Forty-three patients were enrolled: 25 under the OPD group, 10 under the Adm-NoReadmit group, and 8 under the Readmit group. The study flow is presented in Figure 1. The cohort was 53% male (23/43) with a mean age of 66 ± 14 years. Paroxysmal AF was present in 27 patients (63%) and long-standing persistent AF in 16 (37%). Hypertension was the most prevalent comorbidity (36/43, 84%). Baseline characteristics are given in Table 1. Age, sex, AF type, and CHA_2_DS_2_-VASc score did not differ significantly across groups (all *p* ≥ 0.19). Congestive heart failure (CHF) history was substantially more prevalent in the Readmit group (6/8, 75%) than in the Adm-NoReadmit (2/10, 20%) and OPD groups (2/25, 8%; overall *p* = 0.001).

**Table 1:** Baseline clinical characteristics.

Variable	OPD (*n* = 25)	Adm-NoReadmit (*n* = 10)	Readmit (*n* = 8)	*p* (overall)
Age, mean ± SD (years)	66.1 ± 15.3	64.8 ± 14.7	67.0 ± 12.8	0.61
Male sex, *n* (%)	14 (56%)	5 (50%)	4 (50%)	0.88
Hypertension, *n* (%)	22 (88%)	8 (80%)	6 (75%)	0.54
Diabetes mellitus, *n* (%)	5 (20%)	1 (10%)	1 (13%)	0.69
Dyslipidaemia, *n* (%)	16 (64%)	6 (60%)	5 (63%)	0.98
CHF history, *n* (%)	2 (8%)	2 (20%)	6 (75%)	0.001
Prior stroke/transient ischaemic attack (TIA), *n* (%)	5 (20%)	2 (20%)	1 (13%)	0.89
Prior myocardial infarction (MI), *n* (%)	5 (20%)	3 (30%)	0 (0%)	0.21
CHA_2_DS_2_-VASc, median (IQR)	2 (1–4)	2.5 (1–4)	5 (4–6)	0.19
Paroxysmal AF, *n* (%)	18 (72%)	5 (50%)	4 (50%)	0.23
Long-standing persistent AF, *n* (%)	7 (28%)	5 (50%)	4 (50%)	0.23

Fisher’s exact test for categorical variables.

### 3.2 Primary analysis: CMR predictors of 30-day readmission (*n* = 18)

Among admitted patients (*n* = 18; 8 readmitted, 10 not readmitted), several CMR parameters differed between the groups. The results are presented in Table 2 and Figure 2.

**Table 2:** CMR parameters: readmission vs. no readmission among admitted patients (*n* = 18).

CMR parameter	Adm-NoReadmit (*n* = 10)	Readmit (*n* = 8)	*p*-value	AUC (95% CI)	Sens. (%)	Spec. (%)
LA reservoir strain (%)	15.4 (13 to 17)	6.6 (5 to 9)	<0.001	0.963 (0.90–1.00)	88	100
LAVI/LVEF ratio	0.57 (0.50 to 0.65)	1.21 (0.83 to 2.48)	0.002	0.913 (0.74–1.00)	100	80
LAVI (mL/m^2^)	35.7 (30.3 to 39.5)	62.0 (50.1 to 79.1)	0.007	0.850 (0.69–1.00)	88	70
Atrial hyperenhancement (non-dedicated LGE)^a^	0/10 (0%)	8/8 (100%)	<0.001	—^a^	—	—
RVEF (%)	60.0 (52.2 to 65.5)	43.5 (30.5 to 52.5)	0.004	0.838 (0.67–1.00)	88	70
LAEF (%)	49.7 (41.9 to 65.6)	25.0 (23.3 to 26.3)	0.021	0.825 (0.65–1.00)	88	90
RVESVi (mL/m^2^)	29.0 (23.3 to 33.8)	49.5 (36.3 to 80.8)	0.019	0.825	75	80
Non-isch. LV scar	0/10 (0%)	5/8 (63%)	0.009	0.813^b^	63	100
LVEF (%)	63.0 (58.8 to 66.0)	53.5 (33.3 to 60.0)	0.036	0.781	88	70
LV circ. strain (%)	−20 (−22 to −18)	−16 (−20 to −11)	0.050	—	—	—
LV GLS (%)	−16 (−17 to −14)	−14 (−16 to −10)	0.065	—	—	—
LACE score	9.5 (7.0 to 10.8)	11.0 (10.2 to 12.0)	0.125	0.719 (0.44–0.95)	75	70

LACE: AUC 95% CI from bootstrap (5,000 iterations); Sens/Spec at Youden optimal cutpoint ≥11 (LACE ≥10 gives Sens 75%, Spec 50%).

Values are median (IQR) unless otherwise stated. RVESVi, right ventricular end-systolic volume index; LV circ. strain, LV circumferential strain; GLS, global longitudinal strain; Non-isch., non-ischaemic.

a Atrial hyperenhancement was assessed on an LV-optimised protocol not validated for atrial tissue and is reported only descriptively; no discrimination statistic is assigned (— = not applicable).

b Sensitivity/specificity for non-ischaemic LV scar derived from the 2 × 2 table.

**Figure 2:**
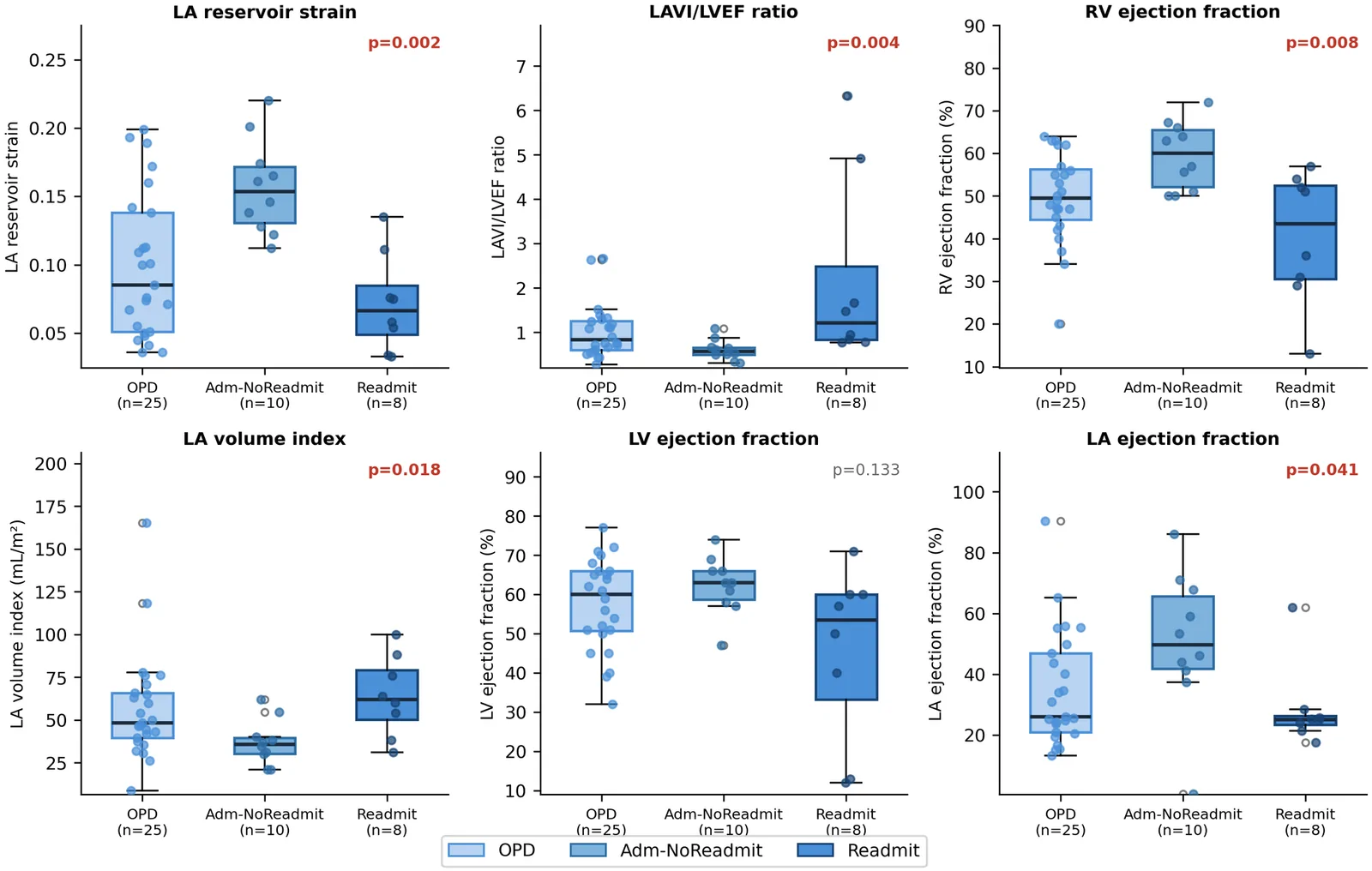
CMR parameters across three clinical groups. The box plots show median (IQR) with individual data points. The Kruskal–Wallis *p*-value is shown per panel.

LA reservoir strain was significantly lower in the Readmit group [median, 6.6% (IQR, 5%–9%)] than in the Adm-NoReadmit group [15.4% (13%–17%); *p* < 0.001] and had the highest AUC among continuous predictors (AUC, 0.963; 95% CI, 0.90–1.00). At the exploratory, internally derived Youden cutpoint of <11.1%, sensitivity was 88% (95% CI, 47%–100%) and specificity was 100% (95% CI, 69%–100%). The Nagelkerke R^2^ for the univariate LA reservoir strain logistic regression model was 0.79. Accounting for selection among the screened continuous predictors, the optimism-corrected AUC was 0.919. In Firth’s penalised regression, each 1-SD (5.5%) decrement in LA reservoir strain was associated with markedly higher odds of readmission (odds ratio [OR], 16.8; 95% CI, 2.6–2,669; penalised-likelihood ratio *p* = 0.0002); the very wide interval reflects the small event count and quasi-complete separation.

The LAVI/LVEF ratio also discriminated well (AUC, 0.913; *p* = 0.002), as did LAVI (AUC, 0.850; *p* = 0.007), RVEF (AUC, 0.838; *p* = 0.004), RVEF (AUC, 0.838; *p* = 0.004). On non-dedicated LGE imaging, qualitative atrial hyperenhancement was present in all eight readmitted patients and in none of the 10 Adm-NoReadmit patients (complete separation); because it was assessed on an LV-optimised protocol not validated for atrial tissue, this finding is reported only as an exploratory qualitative finding and is not assigned a discrimination statistic. The complete 8/8 vs. 0/10 split should not be read as biological evidence of a fibrosis mechanism. Non-ischaemic LV scar was present in 5 of 8 readmitted patients vs. 0 of 10 in the Adm-NoReadmit group (*p* = 0.009).

#### 3.2.1 CMR timing and sensitivity analysis

All 18 CMR examinations in admitted patients were performed before the index admission. The median CMR-to-index admission interval was 134 days (4.4 months; IQR, 48–304 days; range, 16–1,066 days). The interval was longer in the Readmit group (median, 280 days; IQR, 94–413) than in the Adm-NoReadmit group (median, 78 days; IQR, 34–204), although this difference did not reach statistical significance (*p* = 0.237). Fourteen of 18 patients (78%) had undergone CMR within 12 months of the index admission.

In the pre-specified sensitivity analysis restricted to these 14 patients (6 readmission events), LA reservoir strain retained discrimination for 30-day readmission (AUC, 0.938; 95% CI, 0.750–1.000), essentially unchanged from the full-cohort estimate (AUC, 0.963). The LAVI/LVEF ratio (AUC, 0.896) and RVEF (AUC, 0.875) also remained discriminating. These results indicate that the association between impaired atrial reservoir strain and readmission was not driven by temporally remote imaging; this subgroup nonetheless included only 14 patients and six events and remains underpowered.

#### 3.2.2 Causes of index admission and readmission

The cause of the index admission was AF with rapid ventricular response (RVR) in nine patients and heart failure in nine patients. Heart failure as the index admission cause was more frequent among patients who were subsequently readmitted (6/8) than among those who were not (3/10). Among the eight, five (62.5%) were readmitted for AF with RVR and three (37.5%) for bleeding related to anticoagulation. Because bleeding has no direct mechanistic link to the atrial substrate, readmission in this cohort represents a heterogeneous endpoint (Table 3).

**Table 3:** Causes of index admission and 30-day readmission.

Cause	No readmission (*n* = 10)	Readmission (*n* = 8)	Total (*n* = 18)
Index admission cause—AF with RVR	7	2	9
Index admission cause—Heart failure	3	6	9
Readmission cause—AF with RVR	—	5	5
Readmission cause—Bleeding (anticoagulation)	—	3	3

In a *post hoc* analysis restricted to AF-related readmissions (excluding the three bleeding events), LA reservoir strain retained discrimination (AUC, 0.940), indicating that the association was not driven by the bleeding cases. However, the three patients readmitted for bleeding also had low LA reservoir strain (median, 7.5%), and therefore, when they were treated as non-events, the discrimination for AF-specific readmission decreased (AUC, 0.831). This suggests that low reservoir strain identifies broadly higher-risk patients and is not entirely specific to arrhythmic readmission. Among the nine patients whose index admission was AF with RVR (two readmissions), median LA reservoir strain was 5.6% in the readmitted vs. 16.1% in the non-readmitted group, but with only two events, this subset cannot support a discrimination estimate.

### 3.3 Three-group CMR phenotyping

The three-group analysis showed a non-monotonic distribution of LA functional parameters (Table 4, Figure 2). The Adm-NoReadmit group consistently had the highest values for LA reservoir function, whereas the Readmit group had the lowest values.

**Table 4:** Three-group Kruskal–Wallis: key CMR parameters (*n* = 43).

CMR parameter	OPD (*n* = 25)	Adm-NoReadmit (*n* = 10)	Readmit (*n* = 8)	KW *p*	Pattern
LA reservoir strain (%)	8.5 (5–14)	15.4 (13–17)	6.6 (5–9)	0.002	Highest in Adm-NoReadmit
RVEF (%)	49.5 (44.5–56.3)	60.0 (52.2–65.5)	43.5 (30.5–52.5)	0.008	Highest in Adm-NoReadmit
LAVI/LVEF ratio	0.83 (0.60–1.26)	0.57 (0.50–0.65)	1.21 (0.83–2.48)	0.004	Lowest in Adm-NoReadmit
LAEF (%)	25.9 (21.0–46.8)	49.7 (41.9–65.6)	25.0 (23.3–26.3)	0.041	Highest in Adm-NoReadmit
LAVI (mL/m^2^)	48.3 (39.4–65.8)	35.7 (30.3–39.5)	62.0 (50.1–79.1)	0.018	Lowest in Adm-NoReadmit
Atrial hyperenhancement (LGE)	7/25 (28%)	0/10 (0%)	8/8 (100%)	<0.001	Highest in Readmit
LVEF (%)	60.0 (50.8–66.0)	63.0 (58.8–66.0)	53.5 (33.3–60.0)	0.133	NS

KW, Kruskal–Wallis; atrial hyperenhancement compared by Fisher-Freeman-Halton exact test; Adm-NoReadmit highest = non-monotonic peak in Adm-NoReadmit; Adm-NoReadmit lowest = non-monotonic trough. *Post hoc* Bonferroni-corrected (×3). NS = not significant.

LA reservoir strain was 8.5% (IQR, 5%–14%) in the OPD group, 15.4% (13%–17%) in the Adm-NoReadmit group, and 6.6% (5%–9%) in the Readmit group (Kruskal–Wallis *p* = 0.002). *Post hoc* comparisons showed significant differences between the OPD and Adm-NoReadmit (Bonferroni-corrected *p* = 0.004) and between the Adm-NoReadmit and Readmit groups (*p* < 0.001) but not between OPD and Readmit (*p* = 0.239). A comparable pattern was observed for RVEF (*p* = 0.008) and LAEF (*p* = 0.041). The LAVI/LVEF ratio showed an inverted pattern: 0.83 for the OPD group, 0.57 for Adm-NoReadmit, and 1.21 for Readmit (*p* = 0.004). Atrial hyperenhancement prevalence was 28% in the OPD group, 0% in Adm-NoReadmit, and 100% in Readmit (*p* < 0.001).

### 3.4 ROC curve analysis and DeLong comparisons

ROC curves are presented in Figure 3, and diagnostic performance is presented in Table 5. LA reservoir strain had the highest point estimate of AUC among continuous parameters (0.963; 95% CI, 0.90–1.00), with an optimal cutpoint of <11.1%. The LAVI/LVEF ratio (AUC, 0.913; cutpoint ≥0.77: sensitivity, 100%; specificity, 80%) and LAVI alone (AUC, 0.850) also showed good discrimination.

**Figure 3:**
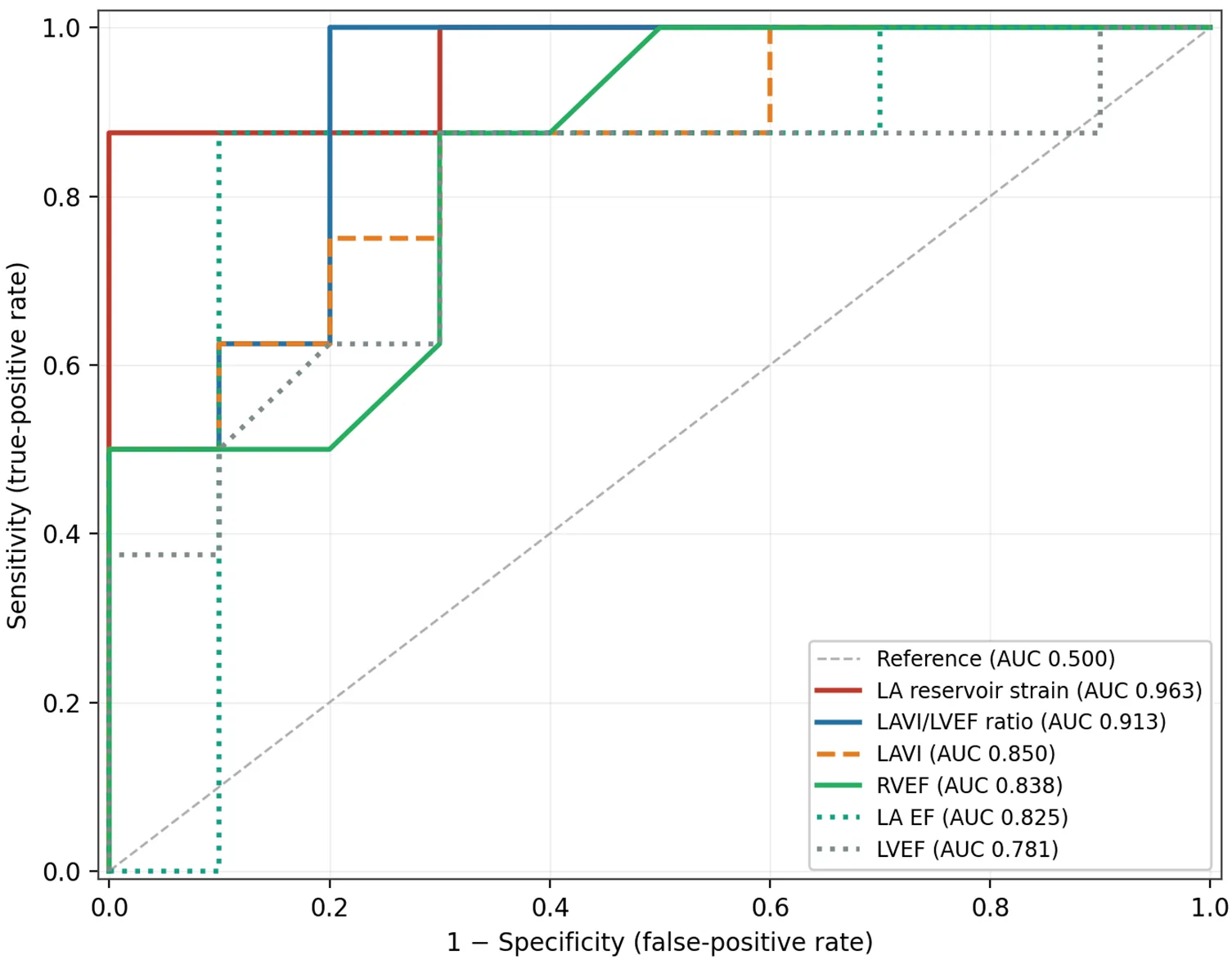
ROC curves for CMR predictors of 30-day readmission after an AF-related index admission (*n* = 18). Six continuous CMR predictors are shown. Qualitative atrial hyperenhancement is not plotted, because it was assessed on a non-dedicated LGE protocol and is not assigned a discrimination statistic. Dashed line = reference.

**Table 5:** Diagnostic performance of key CMR parameters (exact Clopper–Pearson 95% CIs for sensitivity, specificity, PPV, NPV).

Parameter	AUC (95% CI)	Cutpoint	Sens. (95% CI)	Spec. (95% CI)	PPV (95% CI)	NPV (95% CI)
LA reservoir strain (%)	0.963 (0.90–1.00)	<11.1%	88% (47–100)	100% (69–100)	100% (59–100)	91% (59–100)
LAVI/LVEF ratio	0.913 (0.74–1.00)	≥0.77	100% (63–100)	80% (44–98)	80% (44–98)	100% (63–100)
LAVI (mL/m²)	0.850 (0.69–1.00)	≥38.3	88% (47–100)	70% (35–93)	70% (35–93)	88% (47–100)
RVEF (%)	0.838 (0.67–1.00)	<54%	88% (47–100)	70% (35–93)	70% (35–93)	88% (47–100)
LAEF (%)	0.825 (0.65–1.00)	<28.4%	88% (47–100)	90% (56–100)	88% (47–100)	90% (56–100)

PPV, positive predictive value; NPV, negative predictive value. Exact (Clopper–Pearson) 95% CIs. Atrial hyperenhancement is not shown; it was assessed on an LV-optimised protocol not validated for atrial tissue and is reported only descriptively (see Table 2 and Section 2).

Three pairwise AUC comparisons using DeLong's method (10) (Table 6) showed no significant differences: LA reservoir strain (AUC, 0.963) vs. LAVI/LVEF ratio (AUC, 0.913) had ΔAUC = 0.050 and *p* = 0.543, LAVI/LVEF ratio (AUC 0.913) vs. LAVI alone (AUC 0.850) had ΔAUC = 0.063 and *p* = 0.299, and LA reservoir strain vs. LAVI alone had ΔAUC = 0.113 and *p* = 0.283. With only 18 subjects and eight events, the study lacked the statistical power to detect differences, and therefore, null results reflect limited evidence and not true equivalence.

**Table 6:** Pairwise AUC comparisons by DeLong's method (*n* = 18).

Comparison	AUC A	AUC B	ΔAUC (A–B)	*p*-value	Interpretation
LA strain vs. LAVI/LVEF	0.963	0.913	0.050	0.543	Not significant
LAVI/LVEF vs. LAVI alone	0.913	0.850	0.063	0.299	Not significant
LA strain vs. LAVI alone	0.963	0.850	0.113	0.283	Not significant

DeLong's test for correlated ROC curves (10). All comparisons are underpowered at *n* = 18; non-significant results are inconclusive and not an evidence of equivalence.

### 3.5 LACE score performance and comparison with CMR parameters

LACE scores were available for all 18 admitted patients (range, 6–13; mean, 9.7 ± 2.3). The median LACE score was higher in the Readmit group than in the Adm-NoReadmit group [11.0 (IQR, 10.2–12.0) vs. 9.5 (7.0–10.8)], although this difference was not statistically significant (Mann–Whitney *p* = 0.125), indicating a modest effect size and limited statistical power. An ROC analysis showed a LACE AUC of 0.719 (95% CI, 0.44–0.95). Using the published high-risk threshold of ≥10, sensitivity reached 75%, but specificity was only 50%, resulting in a PPV of 55% and an NPV of 71%—meaning approximately half of patients identified as at high risk were not actually readmitted. The Youden optimal cutpoint of ≥11 improved specificity to 70%, while maintaining sensitivity at 75% (PPV, 67%; NPV, 78%).

Every CMR parameter that reached significance in the primary analysis individually exceeded the LACE AUC: LA reservoir strain, 0.963; LAVI/LVEF ratio, 0.913; RVEF, 0.838; and LAVI, 0.850. The bootstrap ΔAUC between LA reservoir strain and LACE was +0.244, but the *p*-value of 0.100 and wide confidence interval (−0.04 to +0.55) highlight that this cohort was underpowered to formally test AUC superiority. The explained variance is more informative: Nagelkerke R^2^ for LACE alone was 0.15 compared with 0.79 for LA reservoir strain—a numerically larger explanatory value for LA reservoir strain; however, their confidence intervals overlapped, and with eight events, this comparison is exploratory and should not be interpreted as established superiority.

An exploratory combined LACE+ LA reservoir strain model produced a higher apparent AUC than LACE alone; however, because there were only eight events, this combined estimate is unstable and almost certainly overfitted. We therefore do not report its net reclassification improvement or interpret it as evidence of incremental value but present it only as an exploratory observation (the combined curve is labelled “exploratory, overfitted” in Figure 4A). Figure 4 plots the LACE score against LA reservoir strain for each admitted patient, with outcomes indicated by marker shape and both optimal cutpoints overlaid. Two readmitted patients had LACE scores of 6 and 8 but LA reservoir strains of 3.3% (below the imaging cutpoint) and 11.1% (at the cutpoint), respectively. Clinical scoring alone would have classified them as low risk at discharge. Conversely, two non-readmitted patients had LACE scores of 11 and 12 but had LA reservoir strains above 16.1%, indicating preserved atrial reserve despite a clinically concerning admission profile. The absolute numbers are too small to draw firm conclusions, but the direction of these discrepancies aligns with the mechanistic argument that atrial substrate and clinical acuity measure different, non-overlapping aspects of readmission risk.

**Figure 4:**
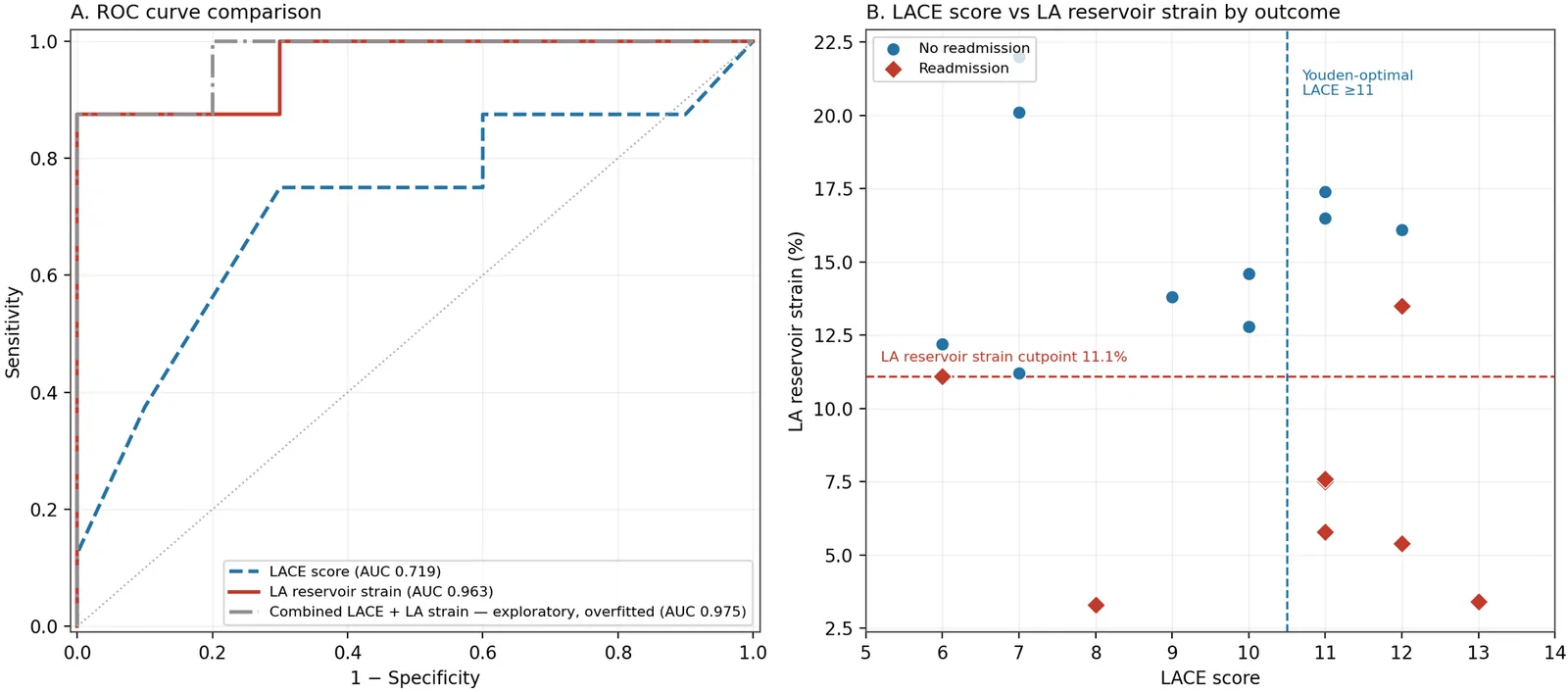
LACE score vs. LA reservoir strain for 30-day readmission prediction (*n* = 18). **(A)** ROC curves for LACE score (AUC 0.719) and LA reservoir strain (AUC 0.963). The combined LACE+ LA reservoir strain model is labelled “exploratory, overfitted” because, with only eight events, it is unstable and not interpreted as evidence of incremental value. **(B)** LACE score (*x*-axis) vs. LA reservoir strain (*y*-axis), stratified by outcome (diamonds = readmitted; circles = not readmitted). The dashed lines indicate the cutpoints (Youden optimal LACE ≥11; LA reservoir strain <11.1%; the published high-risk threshold LACE ≥10 is shown for reference). Two readmitted patients with low LACE scores (6 and 8) had low LA reservoir strain (3.3% and 11.1%), illustrating cases where CMR adds information not captured by clinical scoring alone.

### 3.6 Intercorrelation among CMR predictors

Table 7 presents Spearman rank correlation coefficients among the five key continuous CMR parameters in admitted patients (*n* = 18).

**Table 7:** Spearman rank correlation matrix among key CMR parameters (*n* = 18).

Parameter	LA res. strain	LAVI	LAVI/LVEF	LAEF	RVEF
LA reservoir strain	—	−0.656**	−0.746***	+0.690**	+0.655**
LAVI (mL/m^2^)	−0.656**	—	+0.898***	−0.712***	−0.469*
LAVI/LVEF ratio	−0.746***	+0.898***	—	−0.612**	−0.567*
LAEF (%)	+0.690**	−0.712***	−0.612**	—	+0.448
RVEF (%)	+0.655**	−0.469*	−0.567*	+0.448	—

Values are Spearman *ρ*.

* *p* < 0.05; **p < 0.01; ***p < 0.001.

LA reservoir strain correlated inversely with LAVI (*ρ* = −0.656, *p* = 0.003) and with the LAVI/LVEF ratio (*ρ* = −0.746, *p* < 0.001) and positively with LAEF (*ρ* = +0.690, *p* = 0.002) and RVEF (*ρ* = +0.655, *p* = 0.003). LAVI and the LAVI/LVEF ratio were very strongly intercorrelated (*ρ* = +0.898, *p* < 0.001) as expected because LAVI is the numerator of the ratio. The correlation between LAEF and RVEF did not reach statistical significance (*ρ* = +0.448, *p* = 0.063). The intercorrelations are expected: all five parameters capture different facets of the same disease process, ranging from intact atrial compliance to end-stage fibrotic myopathy. When individual AUC values are interpreted, this shared variance should be kept in mind—the parameters are not independent predictors but rather correlated markers along a single axis of atrial remodelling severity.

### 3.7 Two-group comparison: outpatient vs. admitted (pooled)

To test whether a conventional two-group design (OPD vs. all admitted patients pooled, *n* = 25 vs. 18) could detect CMR differences, we compared all continuous and categorical parameters between groups using Mann–Whitney’s U and Fisher's exact tests. No CMR parameter reached statistical significance (all *p* > 0.05; Supplementary Table S1). CHF history was more prevalent in the pooled admitted group (8% vs. 44%, *p* = 0.009), consistent with Table 1. Among CMR parameters, the nearest misses were sinotubular junction diameter (26.0 vs. 28.5 mm, *p* = 0.060) and the presence of ischaemia on CMR (16% vs. 44%, *p* = 0.083).

Notably, LA reservoir strain (OPD median 9% vs. admitted 12%, *p* = 0.209), LAVI (48.3 vs. 39.1 mL/m^2^, *p* = 0.319), LAVI/LVEF ratio (*p* = 0.675), and atrial hyperenhancement prevalence (28% vs. 44%, *p* = 0.338)—the very parameters that discriminated sharply between readmitted and non-readmitted patients—showed no separation when the two admitted subgroups were pooled. This null result occurred because the Adm-NoReadmit subgroup (with the highest LA reservoir strain of all three groups) arithmetically offset the Readmit subgroup (with the lowest), producing a pooled admitted-group median that resembled the OPD group.

## 4 Discussion

### 4.1 Principal findings

Among admitted patients with AF, CMR-derived LA reservoir strain and the LAVI/LVEF ratio distinguished those readmitted within 30 days from those who were not, with AUCs of 0.963 and 0.913, respectively. These numbers, however, come from 18 patients with only eight events, and overfitting is a real concern. Correcting for predictor selection, the optimism-corrected AUC for LA reservoir strain fell to 0.919, and because the readmitted patients had a markedly higher heart-failure burden, these associations cannot be interpreted as independent, AF-specific effects. A descriptive three-group comparison showed a non-monotonic distribution of LA functional parameters across groups.

### 4.2 LA reservoir strain in AF

We chose the LA reservoir strain over the booster pump or conduit strain for a practical reason: in AF, the atrial contraction phase cannot be reliably identified in the strain curve, and the timing of early diastolic mitral valve opening is equally difficult to pinpoint. In contrast, reservoir strain is defined from LV end diastole to peak LA volume during ventricular systole, a window independent of atrial electrical organisation (5). Nonetheless, reservoir strain magnitude remains dependent on ventricular loading and heart rate (25); cardiac rhythm, heart rate, and complete cardioversion/ablation history at the time of CMR could not be reliably retrieved in this retrospective cohort owing to technical limitations of server-based data extraction, and therefore, unmeasured rhythm and haemodynamic state may have influenced the values (see Limitations).

When reservoir strain is low, the atrial wall has lost its ability to stretch—a sign that the tissue has stiffened, typically from fibrosis, and can no longer absorb the pulmonary venous volume that normally fills the LA during ventricular systole (4). Prior work points in the same direction: Raafs et al. found that LA strain independently predicted prognosis in dilated cardiomyopathy (12), and in the MESA cohort, decreased peak LA strain was associated with incident AF (13). Hopman et al. showed that impaired LA reservoir strain correlated with the extent of LGE-defined atrial fibrosis (5). The present data suggest that the same relationship holds for short-term AF readmission, although the small sample limits how much weight this conclusion can carry.

### 4.3 The LAVI/LVEF ratio

The LAVI/LVEF ratio (AUC, 0.913) combines chronic LA pressure-volume overload and LV systolic impairment. Yilmaz et al. identified it as an independent correlate of 8-year major adverse cardiovascular events (MACE) in STEMI (7), and Aleligne et al. associated it with adverse outcomes in transthyretin amyloidosis (8). We are unaware of prior studies on this ratio for AF readmission. DeLong's test (Table 6) showed a ΔAUC of 0.063 (*p* = 0.299), indicating limited power. Larger datasets are needed to determine whether dividing by LVEF adds value beyond LAVI alone.

The complete separation of qualitative atrial hyperenhancement between the groups—100% in the Readmit group vs. 0% in the Adm-NoReadmit group—is striking but, given the LV-optimised protocol, is reported only as an exploratory qualitative finding and is not assigned a discrimination statistic; this complete split should not be read as biological evidence of an atrial fibrosis mechanism. This finding aligns with the established role of atrial fibrosis in AF maintenance; the DECAAF 5-year follow-up showed that every 10% increase in pre-procedural atrial fibrosis was associated with a 45% increased risk for AF recurrence (6, 14). However, this result has important methodological limitations that prevent drawing strong conclusions. The LGE protocol used in this study was optimised for LV myocardium nulling via a Look–Locker-derived TI, rather than for detailed atrial wall characterisation. The atrial wall averages 2–3 mm in thickness, and standard LV-nulling sequences have limited ability to distinguish the thin atrial myocardium from nearby blood or fat tissue. Dedicated atrial LGE acquisitions—using higher spatial resolution, shorter TIs targeted at atrial tissue, and navigator-gated 3D acquisitions as in the DECAAF protocol—were not performed. Therefore, the binary atrial hyperenhancement variable should be seen as a qualitative indicator based on clinically available LGE images and not as a validated quantitative measure of atrial fibrosis load. A perfect binary split, even with a blinded dual-reader consensus, may reflect protocol limitations as much as true biological differences. A recent narrative review summarises the role and technical requirements of CMR for characterising atrial cardiomyopathy and guiding substrate-based AF ablation, underscoring that dedicated atrial LGE with atrial-targeted inversion times, higher spatial resolution, and respiratory-gated 3D acquisitions is required for reliable fibrosis quantification (15). Future research should use dedicated atrial LGE sequences with quantitative fibrosis staging (e.g., Utah classification) to better characterise the atrial substrate in this setting.

### 4.4 Descriptive differences in LA function across clinical groups

The Adm-NoReadmit group had the highest LA reservoir strain (15.4%), exceeding both OPD (8.5%) and Readmit (6.6%). These are three administratively defined, highly selected groups rather than data-derived phenotypes, and connecting three-group medians does not establish a biological relationship; the following interpretations are therefore offered only as hypotheses:

The OPD group may have stable atrial remodelling that impairs reserve without causing decompensation. The Adm-NoReadmit group may have been admitted for reversible causes (e.g., non-adherence or infection), while retaining preserved atrial reserve. The Readmit group had the lowest reservoir strain, which may reflect more advanced atrial disease; however, this group also had a markedly higher prevalence of heart-failure history (6/8, 75% vs. 2/10, 20%; *p* = 0.001), which may account for the same differences and cannot be separated from an atrial substrate effect given the small number of events.

The key point, if these patterns are consistent in larger groups, is that a patient who has never been hospitalised for AF is not necessarily healthier at the tissue level than one who has. CMR might reveal atrial disease that clinical scoring systems completely miss. This interpretation remains speculative and requires prospective testing.

An alternative explanation for this non-monotonic pattern is referral bias in the OPD group. Outpatients with well-controlled AF usually do not receive CMR, unless there is a suspicion of cardiomyopathy or ventricular dysfunction. Therefore, the OPD group reflects a negatively selected subset of outpatient AF—those with enough concern for CMR—rather than the general stable AF population. This may increase occult atrial myopathy and lower average LA reservoir strain. In contrast, admitted patients often undergo CMR as part of diagnosis, making the Adm-NoReadmit group more representative of the broader admitted AF population.

### 4.5 LACE score and the incremental value of CMR

The LACE score achieved an AUC of 0.719 in this cohort, placing it in the lower range of published estimates for general medical inpatients (usually 0.63–0.74) (11), and was not statistically significant with *p* = 0.125. This lack of significance is probably due to limited statistical power rather than the absence of an effect—however, the modest R^2^ of 0.15 indicates that whatever the true effect size is, it is considerably smaller than that of the CMR parameters. Part of this may stem from limitations inherent in applying LACE outside its original context. The Charlson Comorbidity Index, which provides the C in LACE, does not count AF as a condition, and therefore, two patients with the same overall burden of comorbidities but different AF severities will receive the same Charlson scores. The acuity measure—whether the admission was emergent—adds little discrimination because almost all AF hospitalisations at a tertiary centre are emergency admissions. Similarly, although emergency department (ED) visit count is an important factor for 30-day readmission in heart failure, where patients often go repeatedly through emergency evaluations, patients with AF at our institution frequently bypass the emergency department altogether and are admitted directly through outpatient clinics or cardiology referral pathways.

In this exploratory cohort, the CMR parameters showed numerically higher apparent discrimination than LACE, without demonstrated incremental prognostic value. LA reservoir strain accounted for over five times more variance (R^2^ 0.79 vs. 0.15) and showed a sensitivity–specificity profile that LACE could not match in this sample: 88% and 100%, respectively, compared with 75% and 50% for the published LACE ≥10 threshold. Although the formal AUC difference of +0.244 did not reach *p* = 0.05, the wide and asymmetric confidence interval (−0.04 to +0.55) makes it difficult to determine near-equivalence or substantial superiority. With only 18 patients enrolled, the lack of statistical significance is expected. The appropriate interpretation is that the CMR data are underpowered to establish superiority; the observed difference is hypothesis-generating and requires confirmation in an adequately powered prospective study.

We initially explored a combined LACE+ LA reservoir strain model, but with only eight events, this analysis is unstable and overfitted; therefore, we do not report the net reclassification improvement and draw no incremental-value conclusion from it. The scatter plot in Figure 4 nonetheless illustrates, descriptively, that among patients correctly identified only by CMR and missed by LACE, the common feature was severe atrial functional impairment alongside a LACE score that would not have prompted intensive post-discharge monitoring. This illustrates a conceptual limitation of clinical scores that extends beyond LACE: they record how sick the patient was during the index admission but contain no structural information about why the heart might fail to stay stable after discharge. A high LACE score can arise from a prolonged admission for a reversible precipitant in a patient with intact atrial reserve; the same score can arise from a brief admission in a patient with a functionally exhausted atrial reserve. Those two patients carry very different readmission risks, and imaging may add information that clinical scoring does not capture.

However, none of this addresses the practical issue of when to order a CMR for AF readmission risk. The data suggest that the two sources of information are not redundant. A reasonable clinical workflow might use LACE at discharge as a quick initial screen—easy to calculate from routinely collected data—while reserving CMR-based strain analysis for patients in the uncertain middle range, where a clinical score alone leaves the clinician unsure. Whether this tiered approach actually reduces readmissions without overusing imaging cannot be determined from 18 patients. What can be determined is whether future studies should include both. Based on the evidence here, the answer is yes: including LACE alongside CMR parameters in a prospective cohort would enable a properly powered net reclassification improvement (NRI) analysis and a more rigorous test of whether the ΔAUC of +0.244 observed here is significant.

### 4.6 Limitations

The most important statistical limitation in this study is our inability to perform multivariable regression. With 18 admitted patients and only eight readmission events, the events-per-variable (EPV) ratio is 8 for any single predictor—the minimum threshold for borderline acceptable logistic modelling. Adding a second predictor reduces EPV to 4, well below the established minimum of 10 required for reliable coefficient estimation. Forcing bivariate models in this dataset confirmed this empirically: regression coefficients were grossly inflated (e.g., LAVI/LVEF *β* ≈ 10.1, OR 24,000) and models failed to converge, consistent with quasi-complete separation driven by the near-perfect discriminative performance of LA reservoir strain alone. Furthermore, the CMR parameters were substantially intercorrelated (Spearman *ρ* 0.45-0.90), a consequence of their shared biological origin in atrial myopathy, which would produce collinearity and further destabilise multivariable estimates even with larger samples. All analyses in this study are therefore unadjusted univariate estimates; residual confounding by CHF history (prevalence 75% in Readmit vs. 20% in Adm-NoReadmit, *p* = 0.001) and other clinical variables cannot be excluded and should be addressed in future adequately powered studies. We explicitly recommend that readers treat every reported OR, AUC, and R^2^ value as hypothesis-generating rather than definitive.

Second, the CMR-to-admission interval was longer in the Readmit group (median 9.2 months) than in the Adm-NoReadmit group (median 2.6 months). Because more remote imaging would, if anything, be expected to discriminate less well, this imbalance biases against our finding and does not explain the observed association; nonetheless, it should be considered when interpreting the results. CMR was performed at variable time points relative to the index clinical event (up to 5 years prior). However, the principal CMR parameters driving our results—atrial fibrosis (LGE) and its functional consequence (reduced reservoir strain)—reflect predominantly irreversible structural changes (this stability argument applies chiefly to the fibrosis/LGE findings; the functional parameters are more dynamic, as noted below). Established atrial replacement fibrosis, the type detected by LGE, is widely regarded as irreversible once formed: collagen deposition that replaces necrotic cardiomyocytes creates permanent scar tissue that does not regress spontaneously (16, 17). Burstein and Nattel demonstrated in experimental models that even after restoration of sinus rhythm and normalisation of atrial size, atrial fibrosis and conduction abnormalities persisted (18). Dzeshka et al. concluded in their comprehensive review that myocardial fibrosis was more likely to be slowed down than reversed (19). The DECAAF 5-year follow-up showed that baseline atrial fibrosis remained a significant predictor of AF recurrence over the entire observation period, with every 10% increase in fibrosis burden accounting for a 45% increased risk (14). Similarly, once formed, atrial fibrosis has been described as difficult to reverse (17, 20). These observations support the biological plausibility of a CMR-to-event interval of up to 5 years for fibrosis-related parameters, as the fibrotic substrate is unlikely to have improved substantially between the CMR date and the clinical event. Nonetheless, atrial volumes and strain may change more dynamically with haemodynamic loading conditions, and the 5-year window remains a limitation for these parameters specifically.

Third, in this study, heart-failure history was markedly imbalanced between the groups (6/8, 75%, in the Readmit group vs. 2/10, 20%, in the Adm-NoReadmit group; *p* = 0.001). Because the events-per-variable constraint precludes multivariable adjustment, we cannot separate the contribution of heart failure from that of the atrial substrate, and the CMR parameters cannot be described as independent of heart failure. The CMR findings should therefore be interpreted as markers of a shared structural–functional substrate rather than as independent discriminators.

Fourth, qualitative atrial hyperenhancement was assessed on an LV-optimised protocol (LV-nulling inversion time, two-dimensional, non-respiratory-gated) that is not validated for atrial tissue characterisation and cannot resolve the thin (2–3 mm) atrial wall reliably. Prior catheter ablation or cardioversion may also produce iatrogenic atrial enhancement. Ablation and cardioversion history was incompletely captured because several patients underwent these procedures at other institutions, and complete procedural records were not available; more broadly, cardiac rhythm and heart rate at the time of CMR could not be reliably retrieved owing to technical limitations of the hospital's server-based data extraction, which constrains the biological interpretation of the functional parameters. Atrial LGE is therefore reported only as an exploratory qualitative finding, and the complete between-group separation should not be interpreted as validated fibrosis quantification.

Reproducibility was assessed by a blinded re-measurement of 22 CMR examinations. Intra- and inter-observer agreement was excellent for the continuous parameters: LA reservoir strain (intra-observer ICC 0.998, 95% CI 0.995–0.999; inter-observer ICC 0.952, 95% CI 0.892–0.974), LAVI (both ICC 1.000), and LVEF (intra-observer ICC 0.999, inter-observer ICC 1.000); the inter-observer mean absolute difference for LA reservoir strain was 1.5% (limits of agreement −3.7% to +3.2%). For the binary atrial hyperenhancement read, agreement was almost perfect intra-observer (Cohen's *κ* 0.898, 95% raw agreement) and substantial inter-observer (*κ* 0.794, 90% raw agreement). The inter-observer agreement for LA reservoir strain, although excellent, was lower than the intra-observer agreement (ICC 0.952 vs. 0.998), consistent with the known operator dependence of feature-tracking strain; reproducibility may still be lower in other settings, particularly in atrial fibrillation with beat-to-beat variability.

The retrospective, single-centre design of the study also introduces selection bias: patients who underwent CMR are not representative of all patients with AF, and the findings may not transfer to broader populations. In addition, because outcome ascertainment relied on the single-centre electronic medical record, readmissions to other hospitals could not be captured, which may under-ascertain events and misclassify readmission status.

## 5 Conclusion

In this hypothesis-generating single-centre cohort (the CMRRADAR-AF study), CMR-derived LA reservoir strain (AUC 0.963), LAVI/LVEF ratio (AUC 0.913), and qualitative atrial hyperenhancement on non-dedicated LGE separated readmitted from non-readmitted patients; however, atrial LGE was assessed on an LV-optimised protocol and is reported only as an exploratory qualitative finding. LA reservoir strain discriminated readmission with higher apparent accuracy than the LACE clinical risk score (AUC 0.719). After correction for predictor selection, the optimism-corrected AUC was 0.919, and because the readmitted patients had a higher heart-failure burden, these associations cannot be considered independent of heart failure. In an exploratory combined analysis, adding LA reservoir strain to LACE produced a numerically higher AUC; however, with only eight events, this estimate is unstable and overfitted, and therefore, we do not report reclassification metrics or claim incremental prognostic value. Descriptive differences in LA function were observed across the three administratively defined clinical groups, with a non-monotonic distribution of LA functional parameters; these are presented as hypothesis-generating group differences rather than data-derived phenotypes.

Although these results point towards a role for CMR in risk stratification at discharge, they should be treated as only preliminary. Prospective, multicentre studies with larger enrolment, uniform CMR timing, quantitative fibrosis staging, and direct comparison against clinical risk models are needed before any of these parameters can enter routine clinical practice.

## Data Availability

The raw data supporting the conclusions of this article will be made available by the authors, without undue reservation.

## Supplementary material

Supplementary Table S1 Clinical and CMR parameters in outpatients (OPD, *n* = 25) vs. pooled admitted patients (*n* = 18).
