# Supplementary material for: CMR-derived left atrial reservoir strain and the LAVI/LVEF ratio are associated with 30-day readmission in atrial fibrillation: the CMRRADAR-AF retrospective cohort study
Source: Front Cardiovasc Med. 2026 Oct 20;13:1845659. doi: 10.3389/fcvm.2026.1845659 (PMC13727458; doi:10.3389/fcvm.2026.1845659)
Supplement: Supplementary file 1 [file Table1.docx]

**Supplementary Table S1. Clinical and CMR parameters in outpatients (OPD, n = 25) vs. pooled admitted patients (n = 18)**

| **Parameter** | **OPD (n=25)** | **Admitted, pooled (n=18)** | **p-value** | **Test** |
| --- | --- | --- | --- | --- |
| LA reservoir strain (%) | 8.5 (5–14) | 12 (8–16) | 0.209 | MWU |
| LAVI (mL/m²) | 48.3 (39.4–65.8) | 39.1 (32.1–61.4) | 0.319 | MWU |
| LAVI/LVEF ratio | 0.83 (0.60–1.26) | 0.77 (0.54–1.05) | 0.675 | MWU |
| LAEF (%) | 25.9 (21.0–46.8) | 39.3 (24.9–57.6) | 0.273 | MWU |
| RVEF (%) | 49.5 (44.5–56.3) | 53.0 (50.0–61.5) | 0.309 | MWU |
| LVEF (%) | 60.0 (50.8–66.0) | 60.0 (51.8–65.3) | 0.909 | MWU |
| LV GLS (%) | −13 (−16 to −10) | −15 (−16 to −14) | 0.295 | MWU |
| Atrial hyperenhancement (non-dedicated LGE), n (%) | 7/25 (28%) | 8/18 (44%) | 0.338 | Fisher |
| CHF history, n (%) | 2/25 (8%) | 8/18 (44%) | 0.009 | Fisher |
| Ischaemia on CMR, n (%) | 4/25 (16%) | 8/18 (44%) | 0.083 | Fisher |

*Values are median (IQR) or n (%). MWU, Mann–Whitney U test; Fisher, Fisher’s exact test. Pooled admitted patients comprise all index-admitted patients regardless of readmission status (Adm-NoReadmit + Readmit). Atrial hyperenhancement was assessed qualitatively on an LV-optimised LGE protocol not validated for atrial tissue and is reported descriptively. No CMR parameter reached statistical significance (p < 0.05) in the OPD vs. pooled admitted comparison; CHF history (a clinical variable) was more prevalent in admitted patients, consistent with Table 1.*
